# Neural Correlates of Liberalism and Conservatism in a Post-communist Country

**DOI:** 10.3389/fnhum.2019.00119

**Published:** 2019-04-12

**Authors:** Jan Kremláček, Daniel Musil, Jana Langrová, Martin Palecek

**Affiliations:** ^1^Faculty of Medicine in Hradec Králové, Charles University, Hradec Králové, Czechia; ^2^Philosophical Faculty, University of Hradec Králové, Hradec Králové, Czechia

**Keywords:** error related negativity, political attitude, liberalism, conservatism, neuropolitics

## Abstract

A previous experiment showed that there was a strong correlation between conservatism/liberalism and brain activity, linked to an error response (*r* = 0.59, *p* < 0.001) in the USA political environment. We re-ran the experiment on a larger and age-homogeneous group (*n* = 100, 50 females and 50 males, aged 20–26 years) in the Czech Republic; a European country with a different sociocultural environment and history. We did not find a relationship between the brain activity connected to conflict monitoring and self-reported conservatism/liberalism orientation (ρ = −0.11, *p* = 0.297) or conservatism/liberalism validated for the USA agenda (ρ = −0.01, *p* = 0.910). Instead of replicating the previous study, we decided to test the hypothesis under a different socio-cultural context. Our results support a view of self-reported or validated, conservative or liberal attitudes as a complex behavioral pattern. Such a behavioral pattern cannot be determined with statistical significance, using a simple Go-NoGo detection task, without accounting for confounding factors such as age and socio-cultural conditions. Sufficiently powered studies are warranted to evaluate this neuro-political controversy.

## Introduction

Political orientation significantly affects behavior and decision making on an individual and social level. Increasingly there are attempts to describe the factors that shape political orientation across anthropological directions with political neuroscience (Jost et al., 2014). Neuroscientific methods with objective measurements can verify or generate hypotheses related to the political orientation with conservative stance, characterized by closeness and holding tradition vs. a liberal orientation associated with openness and bringing about a change in the order (Jost and Amodio, 2012). Several studies advocating a strong correlation between neurocognitive settings and political preference continues to increase over time (for review, see Schreiber, 2017).

A recent study (Amodio et al., 2007), hereinafter referred to as Am2007, demonstrated that a person’s self-reported political attitude may be closely linked to a neural correlate accompanying a repeated error response, the error related negativity (ERN), in a simple laboratory detection task. The authors found a statistically significant relationship between the amplitude of the ERN and self-evaluation on the liberalism/conservatism (L/C) axis. They demonstrated that participants who presented a higher degree of liberalism in their answers had a larger ERN amplitude, and this fact was interpreted as a higher sensitivity to incentives for change to established rules.

The ERN represents a negative component of event related potentials (ERP) culminating above the medial-frontal cortex, about 50 ms after the moment of an error response (Gehring et al., 2012). Probable neural origins of ERN are localized in the frontomedial cortex, the anterior and rostral cingulate cortex, and the adjacent supplementary motor cortex (Iannaccone et al., 2015), as was also demonstrated by intracortical recordings (Brázdil et al., 2005). The ERN, characterized as a response to conflict, is behavior- and context- dependent. The ERN changes with several factors, such as personal characteristics, psychopathology, age, condition of neurotransmitters (diet, previous experiences) or relationship to the task being performed (Gehring et al., 2012; Larson et al., 2014). As is the case for other ERP, ERN is sensitive to the various aforementioned factors; however, its behavioral interpretation is difficult, as an undistinguishable ERN change may be caused by different conditions. Therefore, we found the results explaining political ideology, by considering ERN (Amodio et al., 2007; Jost and Amodio, 2012; Weissflog et al., 2013; Jost et al., 2014) appealing, and we decided to test if the results are robust with respect to different sociocultural environments. We attempted to replicate the results of the previous Am2007 study in the Czech Republic—a democratic country with a communistic history (1948–1989), in which self-evaluation of L/C need not correspond to the general political orientation. To account for different environmental and historical conditions, we calibrated political preferences according to the values associated with a typical agenda of US liberalism and conservatism in addition to the self-report. We expected to verify the extent to which the original hypothesis of correlation between neurological setting and political preference is specific to the self-reported L/C and US environment.

## Materials and Methods

### Study Cohort and Power Analysis

In our study, we attempted to avoid certain known biasing factors such as age, social strata or sex, which may be related to political orientation. We examined a group of 100 volunteers. At the same effect size (*r*~0.50), as that described by Amodio et al. (2007); who tested 43 volunteers, the power of our test would be 99.9%, for half of the effect (*r*~0.25) the power will be still 71.4% (Cohen, 1992). Doubling the group size should eliminate the “winner’s curse” effect (Button et al., 2013).

One-hundred university students (50 females and 50 males) aged 20–26 years (median 22.7; lower quartile 21.7; upper quartile 23.6 years), without health complaints, not being treated for neurological, psychiatric or ophthalmological pathologies and without dependency on smoking, gambling, marijuana or alcohol, were selected by means of an internet questionnaire.

### Questionnaire Investigation of Political Orientation

Before electro-physiological examination, each participant completed a questionnaire in a separate room in order to determine his/her political orientation. For self-evaluation, participants ranked their political orientation on a scale from absolute liberalism (0) to pure conservatism (10). To validate their self-assessment, the questionnaire also contained 22 questions related to crucial criteria for L/C political orientation: (1) resisting vs. advocating social change; and (2) accepting vs. rejecting inequality (Jost et al., 2014). For each question, we offered two opinions, one representing a liberal opinion and another a conservative political framework, though these terms did not appear in the opinions directly. The polarity of views was randomly mixed in order to limit designation of one side of the scale according to the self-evaluation on a general level. One additional question was a verbal numerical task to test participants’ attention.

Participants were informed that there was no time limit for completing the questionnaire. After completion, the participant sealed his/her questionnaire in an envelope, which was opened after the evaluation of all the electrophysiological data (i.e., from all 100 individuals).

The questionnaire opinions concerned issues of gay marriage, abortion, the death penalty, social policy etc. We converted opinions related to the authorities of the federal government of the United States to an analogous issue of the transfer of competences from the national level of individual member states to EU bodies. The Czech Republic is a classic example of a non-confessional society^1^, and for this reason, we did not include opinions relating to religious values.

The opinions and their weights are listed in Table 1. In addition to the L/C orientation, we also determined the level of Openness, Traditionalism (Altemeyer, 1996), and Equality (Kluegel and Smith, 2017), as these values have also been reported as related to error monitoring (Weissflog et al., 2010, 2013).

**Table 1:** Questionnaire for investigation of political orientation: opinions and their weights.

			Weight of the 1st opinion for:			
	1st opinion	2nd opinion—opposite to the 1st	Liberalism	Openness^a^	Traditionalisms	Equality^b^
1.	Citizens with higher incomes should contribute more to the state budget.	All citizens should contribute the same amount to the state budget, regardless of their income.	1			1
2.	Costs for healthcare should be covered primarily by the individual.^c^	Costs for healthcare should be covered primarily by the state.	−1			1
3.	I am in favor of keeping the law on registered partnership. Persons of the same sex should be allowed to marry.	Registered partnership should be abolished. Persons of the same sex should not be allowed to marry	1	1	−1	1
4.	Control point, please draw a ring around the number which is the sum of 2 + 1	Control point, please draw a ring around the number which is difference 10 − 7				
5.	Homosexual couples should not be allowed to adopt a child from a children’s home.	Homosexual couples should have the right to adopt a child from a children’s home.	−1	−1	1	−1
6.	Education should be financed from the state budget.^d^	Education should be financed primarily by students and the private sector.	1			
7.	Economic growth should have priority over environmental protection.	Economic growth should not be supported at the expense of environmental protection (Wilson and Patterson, 1968; Conover and Feldman, 1981; Hunter, 1991; Altemeyer, 1996; Jost et al., 2009; Jost, 2009; Soenens and Duriez, 2012; Weissflog et al., 2013; Bell, 2014; Cichocka et al., 2016; Nielsen, 2016; Kluegel and Smith, 2017).	−1			
8.	The state should actively support the largest number of socially disadvantaged citizens.	The state should not interfere in the life of citizens any more than is essentially necessary.	1			
9.	The Czech Republic should take in refugees from countries where the dominant religion is Islam.	The Czech Republic should not take in refugees from countries where the dominant religion is Islam.	1	1	−1	1
10.	I find displays of sexuality in the media acceptable.	I find displays of sexuality in the media unacceptable.	1	1	−1	1
11.	I am in favor of at least partial controls on internet content.	Internet content should never be subjected to any controls.	−1	−1	1	−1
12.	Smoking in public areas should be limited.	Smoking should not be limited.	1			
13.	Success is determined by several factors, which individuals do not have entirely under their own control.	The success of an individual depends primarily on his/her own efforts.	1			
14.	The Czech Republic should not accept EU dictates.	The Czech Republic should co-operate with the EU also in the form of adopting central laws.	−1			
15.	I agree with restitution to the church.	I do not agree with restitution to the church.^e^	1			
16.	I would find it acceptable to have members of the Roma community as my neighbors.	I would not like to have members of the Roma community as my neighbors.	1	1	−1	1
17.	The extraction limits for the Czech Republic should not be exceeded.	The Czech Republic should exceed extraction limits within its own territory.	−1			
18.	I am against the teaching of sex education in basic schools.	I am in favor of the teaching of sex education in basic schools.	−1	−1	1	−1
19.	The state should allow severely ill people to decide in favor of euthanasia.	The state should not allow severely ill people to decide in favor of euthanasia.	1	1	−1	1
20.	The possibility of gun ownership should be more strictly controlled by the state.	Every citizen has the right to own a gun for his/her own personal protection.	1			
21.	A pregnant woman should have the right to decide to terminate her pregnancy.	Abortion should be prohibited by the state.	1	1	−1	1
22.	People with a communist past should have the same right to hold high political office as others.^f^	People with a communist past should be barred from high political office.	−1			
23.	Racial discrimination is the main cause why the majority of the Roma population are unemployed.	Roma who are unemployed are mostly to blame for their situation.	1	1	−1	1

*^a^Openness (Buttrick and Oishi, 2017; Kluegel and Smith, 2017). ^b^Equality (Stanley et al., 2017). ^c^This figure represents an openness toward the other fellow citizens. The higher rate of empathy motivates toward higher tendency to share all material goods with the others. This kind of openness is not displayed of amount of charity in the Czech Republic due to the welfare system. This figure is rather displayed by the rate of agreement with wider redistribution of sources from the national budget. ^d^Liberals argue that system of education supposed to be open and free of charge for so many as possible. On the contrary, conservatives argue in general that fee for education improve its value. The education system supposed to be open for those only who want to financially participate on it. ^e^Churches Restitution Act has been passed by the Czech national assembly in 2012 to compensate injustice of communist regime that deliberately plundered churches property, based on the Act 218/1949 on State Economic Support for Churches and Religious Societies. The churches restitution law came so late after the Velvet revolution (1989) because of its controversy. Conservatives oriented nationalistically partially agree with the past-regime approaches toward churches (especially toward the Catholic Church). Czech nationalism has been anti-clerically oriented since at least 1848 (Lass, 1988). Political parties representing their nationalistically oriented electoral do not agree with the Churches Restitution Act and try to revise it. The Cristian Democrats (KDU-CSL) are the exception among conservative parties due to their alignment with the Catholic Church. ^f^This statement is about members of the communist party before the Velvet Revolution in 1989. The reason that all pre-1989 members of the communist party were blacklisted from designated public offices was due to their duty to actively cooperate with the Czechoslovakian secret police (StB) and Soviet secret police (KGB) that was a mutual part of their party membership. The so-called Lustration Law was passed in 1991 and expired in 2000. Nevertheless, there is still widespread public agreement of preventing subjects of this law from formerly designated public offices because they might still be a threat for liberal democracy. There is a justified presupposition that they might still work against the interests of the European Union and NATO. It follows that liberals and democrats are against their presence within key political positions. On the other hand, there are members of the newly formed Communist Party of Bohemia and Moravia (KSČM; its policy is nationalistic and far-conservative), populists, and members of the national far-right that are against the exclusion of pre-1989 communists from political life*.

We used scores from 0 to 10 intentionally, in order to avoid an association between positive and negative values and L/C, especially during the self-assessment. Subsequently, we deducted five points from the reported values in order for the scale to correspond to the Am2007.

### Neurophysiological Examination

#### Stimulation

**Figure 1:**
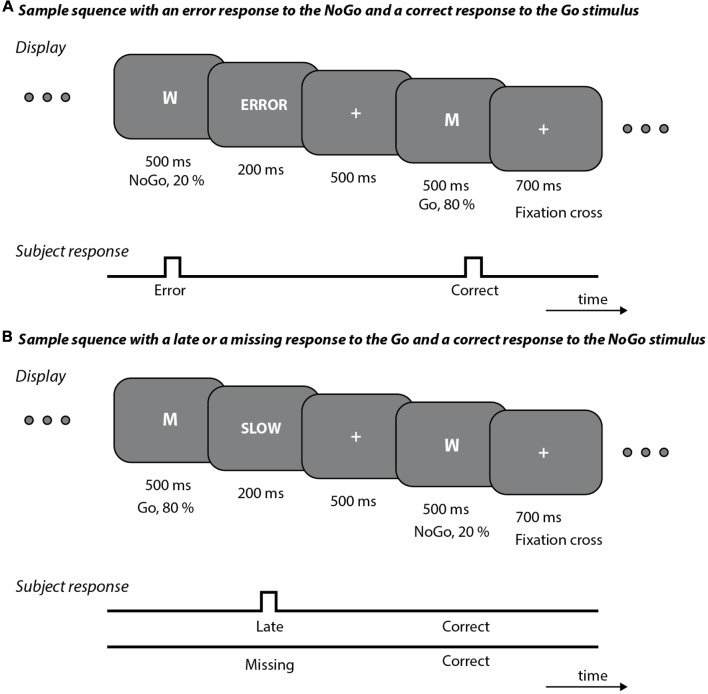
Examples of stimulation sequence and response during electrophysiological examination. **(A)** An incorrect response to the NoGo stimulus was followed by a display of the word ERROR for 200 ms, followed by a fixation cross for 500 ms. The Go stimulus is then depicted, followed by a correct response and by the display of fixation cross for 700 ms. **(B)** An incorrect response (a late or a missing response) to the Go stimulus was followed by the display of the word SLOW, which after 200 ms was replaced by the fixation cross (displayed for 500 ms). In response to the next NoGo stimulus there was, correctly, no button press, and when the stimulus disappeared the fixation cross was displayed for 700 ms.

To the maximum possible extent, the design of our study replicated the method of measuring ERN as implemented in Am2007. For a period of 500 ms, the symbols “M” and “W” were presented on the monitor screen with a probability of 20% and 80%. The participant’s task was to respond to the frequent stimulus by pressing the button (Go) and to ignore the rarer stimulus (NoGo). If the participant did not respond within an interval from 100 to 500 ms after the stimulus, the word SLOW (presented in Czech as POMALÉ) was displayed on the screen for 200 ms. In the case of a response to the NoGo stimulus, the word ERROR (presented in Czech as CHYBA) was displayed again for 200 ms. Each stimulus was preceded by the display of a fixation cross for a period of 700 ms in response to a correct reaction and 500 ms if the aforementioned error message was displayed, see Figure 1.

Five-hundred stimuli were administered in two sessions, separated by an interval of 2 min. The symbols for Go and NoGo were changed between the sessions. The entire test lasted for less than 15 min. The stimuli on the screen were viewed by the participant at a distance of 80 cm, and the Go and NoGo stimuli subtended 2.5°, and the fixation cross subtended 0.6°. The stimuli were presented on a 21-inch computer monitor (HP p1230, USA). The monitor was driven using ViSaGe MKII (Cambridge Research Systems Ltd., Rochester, UK).

#### ERN Registration

During ERN registration we also adhered to the Am2007 settings with the exception of the number of registered scalp locations. Am2007 registered the electroencephalogram (EEG) from 29 derivations but the evaluation of the relationship between political orientation and ERN was conducted only for the FCz localization, and for the N2 peak a response registered from the Cz electrode was used. In our experiment, we recorded EEG from FCz, Cz, A2 derivations, an electrooculogram from two electrodes above the right eye and at its canthus, a referential electrode was on the left earlobe A1. EEG was registered in the frequency band 0.3–100 Hz at a sampling frequency of 1,024 Hz (TrueScan EEG, Alien s.r.o., Hronov, Czech Republic) in a Faraday cage with the background brightness at approx. 1 cd/m^2^.

#### ERN Evaluation

For the ERN, EEG epochs were selected with a length of 800 ms symmetrically around the moment of response to the NoGo stimulus. The epochs were filtered within the range of 1–15 Hz and the DC voltage component, calculated as an average value from the interval −400 to −50 ms, was deducted. Epochs, in which absolute amplitude exceeded 50 μV in any of the registered derivations, were rejected. Following the Amodio study, the remaining epochs were averaged and from this average, the waveform of correct Go responses was subtracted. The ERN was evaluated as the minimal amplitude within an interval of −50 and 150 ms in the FCz derivation.

#### N2 Evaluation

For the N2, EEG epochs of 1,000 ms were selected, starting 200 ms before the NoGo stimulus was displayed. Only epochs with correct NoGo responses were retained and filtered within the range of 1–15 Hz. The DC voltage was deducted from epochs; calculated as an average value from the section −200 to −100 ms. Epochs in which absolute amplitude exceeded the value of 50 μV, in any of the registered derivations, were rejected. The remaining epochs were averaged and N2 was evaluated as the minimal amplitude within an interval of −200 and 400 ms in the Cz derivation.

### Statistical Analysis

The data were statistically processed with R software version 3.4.3 (R Development Core Team, 2018), using the “nortest,” “psych,” “pwr,” and “ggplot2” packages and Matlab version rel. 2018a (Mathworks, Natick, MA, USA).

#### Correlation Analysis

Because the data did not have a normal distribution, we calculated the degree of the relationship between the observed parameters using a Spearman’s rank correlation coefficient.

#### Power Analysis

The group size was determined based on the previous study (Amodio et al., 2007) using R software version 3.4.3 (R Development Core Team, 2018) with “pwr” package.

## Results

### Behavioral Data

On a scale from −5 (absolutely liberal) to 5 (maximum conservative) the median and interquartile range of L/C self-evaluation was −1.0 and (−2.0, 1.3), respectively, in our group (*n* = 100). To compare our results to the original Am2007 study, we read 42 L/C values from their Figure 1A (Amodio et al., 2007). The Am2007 reported self-evaluation of L/C was more liberal than that of our participants (*p* < 0.001) with a median of −2 and both quartiles (−3.0; 0.0).

Beyond the framework of the Am2007 study, we determined value preferences linked to an agenda based on typical values attributed to L/C in the US and calculated a validated L/C orientation. The validated L/C was related to the self-evaluation (Spearman ρ = 0.30, *p* < 0.05), however, it was more liberal (median −0.9, and interquartile range −1.4; −0.4) than the self-evaluation (*p* < 0.001).

Within the electrophysiological part of the examination, the median of participants’ reaction time was 266.9 ms with an interquartile range from 281.8 to 313.3 ms in response to the Go stimuli. The error reaction time to the NoGo stimuli had the median 249.1 ms and the interquartile ranged from 231.7 to 261.2 ms. The median of accuracy for the NoGo condition was 0.75 and 0.97 for the Go condition. In each condition, the accuracy was evaluated as the number of correct responses divided by all possible correct responses.

### ERP Data

**Figure 2:**
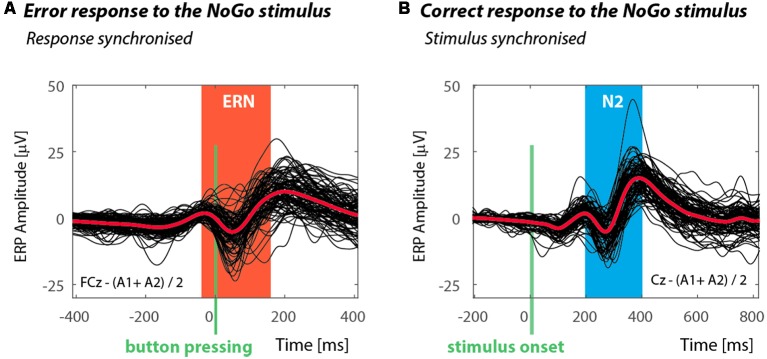
Event related potentials (ERP) responses of individual participants in experiment. **(A)** Black curves show the ERP responses of 95 participants and represent the difference between an incorrect response to the NoGo and a correct response to the Go stimulus. The red curve is the group average, and the red rectangle indicates the interval in which the minimum error related negativity (ERN) value was automatically detected. The green vertical line indicates the moment of pressing the button. **(B)** The graph shows the responses of 95 participants with a correct response to the NoGo stimulus. The interval for automatic detection of the N2 is indicated by the blue rectangle, the green line indicates the time of stimulus onset.

A minimum of six epochs ensures sufficient intra-individual stability for ERN estimation (Olvet and Hajcak, 2009)^2^^,^^3^. ERN curves evaluated at FCz linked to the error response following the NoGo stimulus are displayed in Figure 2A. The median ERN was −9.7 μV and the interquartile range was −13.7 to −6.5 μV. The amplitude of the negative N2 NoGo component registered upon a correct NoGo response at Cz derivation had a −5.0 μV median value and the interquartile ranged from −8.8 to −2.4 μV. The individual curves and their grand average can be seen in Figure 2B.

**Figure 3:**
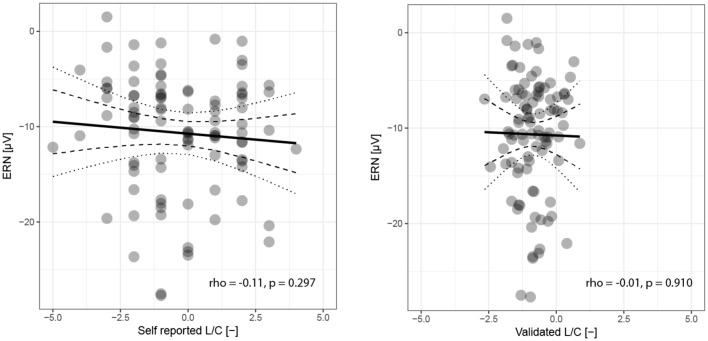
Relationship between amplitude of ERN component and subjective or validated political orientation. Each participant in the experiment is represented by a single point, the horizontal position states subjectively perceived (left plot) or validated (right plot) orientation on a relative scale from full liberalism (−5) to pure conservatism (+5). The vertical co-ordinate corresponds to the ERN component amplitude. The black solid line represents a linear regression with confidence intervals of the estimate depicted by dotted (99.9%) and dashed (95%) curves.

The Spearman’s rank correlation coefficient between ERN and L/C orientation did not show any significant connection either for the values determined by self-evaluation (ρ = −0.11, *p* = 0.297) or for the validated values (ρ = −0.01, *p* = 0.910), see Figure 3.

**Figure 4:**
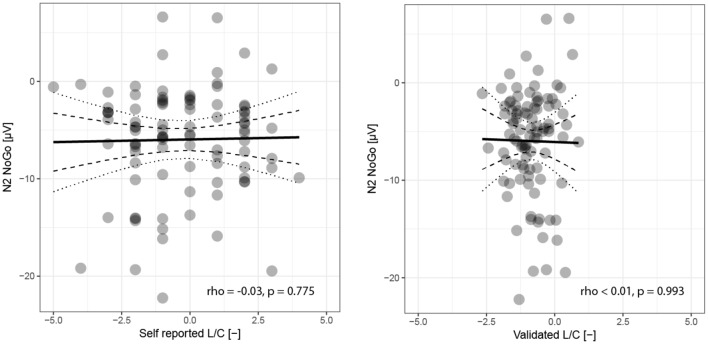
Relationship of amplitude of N2 NoGo component to self-reported and validated political orientation. Each participant in the experiment is represented by a single point, the horizontal position states subjectively perceived (left plot) or validated (right plot) orientation on a relative scale from full liberalism (−5) to pure conservatism (+5). The vertical co-ordinate corresponds to the N2 NoGo component amplitude. The black solid line represents a regression with confidence intervals of the estimate depicted by dotted (99.9%) and dashed (95%) curves.

Similar to the original study, we evaluated the amplitude of the negative component at Cz derivation registered upon a correct NoGo response (N2 NoGo) −5.0 (−8.8, −2.4) μV (see Figure 2B). In this case we did not find a significant relationship to L/C self-evaluation (ρ = −0.03, *p* = 0.775) or its validated variant (ρ < 0.01, *p* = 0.993), see Figure 4.

In addition to the aforementioned parameters, we evaluated ERN- and N2-like components upon a correct response to the Go stimulus. Altogether, these components along with the reaction time and the accuracy of reactions, we conducted the correlation analysis with the behavioral parameters: L/C self and validated orientation, Openness, Traditionalism and Equality. For all 40 comparisons, the ρ ranged from −0.15 to 0.21. After Holm’s correction for multiple comparisons, no significant relationship was observed.

## Discussion

A neural correlate of error response in a simple perception task appeared to be a promising parameter to explain a self-reported L/C orientation. Amodio et al. (2007) described a strong relationship between self-reported L/C and the ERN component (*r* = 0.59, *p* < 0.001) and a similar association to the N2 NoGo component (*r* = 0.41, *p* < 0.01). Contrary to the original Am2007 study, we found no relationship between the ERN or N2 NoGo and L/C orientation based on the self-evaluation in the larger homogenous cohort of 100 participants.

Because L/C represents a broad concept, ranging from sexual agenda to religious or economic issues, it has a variable meaning within an international context (Scruton, 1980; Conover and Feldman, 1981; Bell, 2014). An apparent reason why we were not able to replicate the Amodio’s study could be a different sociocultural environment with a diverse meaning of the L/C concept. To fit the US meaning of L/C to the Czech Republic conditions, we directly evaluated preferences of our participants in light of values traditionally associated with political conservatism and liberalism (Wilson and Patterson, 1968; Inglehart, 1977; Scruton, 1980; Conover and Feldman, 1981; Skitka and Tetlock, 1993). In spite of our validation of the L/C orientation to the traditional agenda, we did not find any relationship with electrophysiological markers of the conflict monitoring or error reactions. Amodio et al. (2007) verified self-reported L/C against preferences during the USA 2004 presidential election, for 21 out of 42 voting participants, they found a strong correlation (*r* = 0.79).

Another possible factor behind the contradictory results might be in the age profile of the groups. Formerly it was demonstrated that the ERN amplitude drops with age (Nieuwenhuis et al., 2002), and in certain directions, the political orientation shifts with age towards conservatism (Danigelis et al., 2007; Cornelis et al., 2009; van Hiel and Brebels, 2011; Soenens and Duriez, 2012; Tilley and Evans, 2014). If a random population sample with a normal age distribution is evaluated a “spurious relationship” between ERN and political opinion could be found, simply because of the hidden age factor. In our study, we eliminated this influence by narrowing the age span of the cohort of participants. If age is the key factor, then the relationship between L/C and ERN should disappear in an age-homogenous group. This is exactly what we observed. The Am2007 study did not describe the age of its cohort. To verify this hypothesis, a group of older Czech citizens should be examined.

A study with a similar power (34 participants, two males, 18–39 years) as Am2007 was conducted among university students in Canada (Weissflog et al., 2010, 2013). In agreement with our results, the study of Weissflog et al. (2013) did not confirm the statistically significant relationship between ERN and the L/C (*r* = 0.27, *p* = 0.13). However, it showed a trend parallel to Am2007 and a significant relationship of the ERN^4^ and N2 markers to Egalitarianism and Right-wing Authoritarianism. All the effects reported, in a direction of a more liberal attitude with openness to changes and support for equality, were connected with the more negative ERN or N2 or a higher accuracy during the test. In their previous conference article, these same authors (Weissflog et al., 2010) also reported that Resistance to change, Dogmatism or Machiavellianism did not appear to have any relationship to conflict monitoring. Based on their conclusions we evaluated Traditionalism, Openness and Equality (see “Materials and Methods” section) *post hoc*, and found that they did not show any relationship to the self-reported or validated L/C orientation. We did not find any relationship among accuracy in the Go or NoGo task and the pursued parameters of political attitude.

Our findings are consistent with other experiments that have previously been performed. Wendell (2016), in a repeat of the original Amodio study (Amodio et al., 2007) using 51 non-student adults (age 24–52 years, 32 females 19 males), revealed a robust null finding (Wendell, 2016, p. 177) when the correlation of the self-reported L/C orientation to the ERN was 0.136 (*p* > 0.05) and −0.122 to the N2 (*p* > 0.05).

There are only two studies that Jost and Amodio (2012) use as examples that support their theory. The first experiment that resembles Amodio’s experiment was provided by Weissflog et al. (2013) as mentioned above. The second study was published by Inzlicht et al. (2009). They studied the correlation between conflict and religious belief, based on a Stroop task using a Go/No-Go task. Jost and Amodio refer to Inzlicht’s study because it was done in relation to religious beliefs that are closely linked to political and cultural conservativism. According to them, Inzlicht’s experiment showed that religious believers (i.e., conservatives) have a significantly weaker ERN response then atheists (i.e., liberals). However, Inzlicht et al. (2009) did not find any link between ERN amplitude and conservativism (*r* = 0.1) or openness (*r* = 0.30) in a relatively small group of students (age 19 ± 1.4 years, 13 females 9 males). These results are in agreement with our study.

We further agree with Wendell, who finds the results of Inzlicht’s experiment open to dispute (Wendell, 2016). According to him the study contained the following problems: first, they did not find any correlation between religious belief and political conservativism; second, contrary to Amodio and Jost’s prediction (Amodio et al., 2007; Jost and Amodio, 2012), religious believers improved their accuracy during the task; third, Inzlicht’s findings did not find any cognitive rigidity among religious believers and this is also a direct contradiction to Amodio and Jost’s presupposition. We claim—to recall our findings and those of Wendell’s direct replication study (Wendell, 2016)—that despite the fact that Amodio’s (Amodio et al., 2007) experiment has been cited more than 500 times it has not yet been successfully replicated.

Although it may be tempting to explain the complex ideology of L/C orientation through a limited number of parameters (Jussim et al., 2016), our examination, conducted on a large, homogeneous study cohort, did not support this hope or approach, and left an open question as to whether political, as well as social functions are the result of a synthesis of a larger number of factors (Vittorio Caprara et al., 2006; Gerber et al., 2010; Wendell, 2016). *Our results urge for other ERN experiments with sufficient power and controlling for age, socio-cultural context and L/C validation*.

## Ethics Statement

For the study we obtained the consent of the Ethical Commission of the University Hospital in Hradec Králové and the work conforms with the 1964 Helsinki Declaration and its later amendments or comparable ethical standards. Each participant was familiarized with the conditions of the study, and informed written consent was obtained from all individual participants included in the study.

## Geolocation Information

The study was conducted in Czech Republic.

## Author Contributions

JK, MP, DM, and JL prepared the experiment and wrote the article. MP and DM designed the political attitude evaluation. JK analyzed the data. JL and DM collected the data.

## Conflict of Interest Statement

The authors declare that the research was conducted in the absence of any commercial or financial relationships that could be construed as a potential conflict of interest.

## Footnotes

^1^Overall religious affiliation of the population of the Czech Republic is as follows: undeclared 45.5%, atheist 34.2%, Roman Catholic 10.3%, other 9.4%, Protestant 0.8% (population by religious belief and by municipality size groups [online]. Czech Statistical Office, https://www.czso.cz/csu/czso/home).

^2^This requirement shrunk our group by five participants to 95 persons. If we raised the minimum number of epochs to 10, the number of participants would be reduced to 84, and in the case of 20 epochs only 24 persons would remain. Through the selection of six epochs, we lessened the elimination of participants who responded with a high degree of precision.

^3^Am2007 does not state how many epochs were averaged for ERN.

^4^Weissflog et al. (2013) reported a residual ERN, which was an ERP difference equally distributed around zero and therefore their interpretation: “conservatives with a preference for tradition could be characterized as having a less responsive ACC” might be too strong with regards to this component.
